# PARIS and SPARTA: Finding the Achilles’ Heel of SARS-CoV-2

**DOI:** 10.1128/msphere.00179-22

**Published:** 2022-05-19

**Authors:** Viviana Simon, Vamsi Kota, Ryan F. Bloomquist, Hannah B. Hanley, David Forgacs, Savita Pahwa, Suresh Pallikkuth, Loren G. Miller, Joanna Schaenman, Michael R. Yeaman, David Manthei, Joshua Wolf, Aditya H. Gaur, Jeremie H. Estepp, Komal Srivastava, Juan Manuel Carreño, Frans Cuevas, PARIS/SPARTA Study Group,, Ali H. Ellebedy, Aubree Gordon, Riccardo Valdez, Sarah Cobey, Elaine F. Reed, Ravindra Kolhe, Paul G. Thomas, Stacey Schultz-Cherry, Ted M. Ross, Florian Krammer

**Affiliations:** a Department of Microbiology, Icahn School of Medicine at Mount Sinai, New York, New York, USA; b Division of Infectious Diseases, Department of Medicine, Icahn School of Medicine at Mount Sinai, New York, New York, USA; c The Global Health and Emerging Pathogens Institute, Icahn School of Medicine at Mount Sinai, New York, New York, USA; d Center for Vaccine and Immunology, University of Georgia, Athens, Georgia, USA; e Department of Infectious Diseases, University of Georgia, Athens, Georgia, USA; f Department of Medicine, David Geffen School of Medicine at UCLA, Los Angeles, California, USA; g Lundquist Institute for Biomedical Innovation at Harbor-UCLA Medical Center, Torrance, California, USA; h Department of Pathology and Laboratory Medicine, David Geffen School of Medicine at UCLA, Los Angeles, California, USA; i Department of Pathology, Medical College of Georgia, Augusta University, Augusta, Georgia, USA; j Department of Medicine, Medical College of Georgia, Augusta University, Augusta, Georgia, USA; k Department of Restorative Sciences, Dental College of Georgia, Augusta University, Augusta, Georgia, USA; l Department of Microbiology and Immunology, University of Miami Miller School of Medicine, Miami, Florida, USA; m Department of Pathology & Immunology, Washington University School of Medicine, St. Louis, Missouri, USA; n Andrew M. and Jane M. Bursky Center for Human Immunology and Immunotherapy Programs, Washington University School of Medicine, St. Louis, Missouri, USA; o Center for Vaccines and Immunity to Microbial Pathogens, Washington University School of Medicine, St. Louis, Missouri, USA; p Department of Epidemiology, School of Public Health, University of Michigan, Ann Arbor, Michigan, USA; q Department of Ecology and Evolution, University of Chicago, Chicago, Illinois, USA; r Department of Infectious Diseases, St Jude Children’s Research Hospital, Memphis, Tennessee, USA; s Department of Pathology, Molecular and Cell-Based Medicine, Icahn School of Medicine at Mount Sinai, New York, New York, USA; t Center for Vaccine Research and Pandemic Preparedness (C-VARPP), Icahn School of Medicine at Mount Sinai, New York, New York, USA; u Department of Pathology, University of Michigan, Ann Arbor, Michigan, USA; University of Saskatchewan

**Keywords:** COVID-19, SARS-CoV-2, antibodies, cohort study, reinfection

## Abstract

To understand reinfection rates and correlates of protection for severe acute respiratory syndrome coronavirus 2 (SARS-CoV-2), we established eight different longitudinal cohorts in 2020 under the umbrella of the PARIS (Protection Associated with Rapid Immunity to SARS-CoV-2)/SPARTA (SARS SeroPrevalence And Respiratory Tract Assessment) studies. Here, we describe the PARIS/SPARTA cohorts, the harmonized assays and analysis that are performed across the cohorts, as well as case definitions for SARS-CoV-2 infection and reinfection that have been established by the team of PARIS/SPARTA investigators.

**IMPORTANCE** Determining reinfection rates and correlates of protection against SARS-CoV-2 infection induced by both natural infection and vaccination is of high significance for the prevention and control of coronavirus disease 2019 (COVID-19). Furthermore, understanding reinfections or infection after vaccination and the role immune escape plays in these scenarios will inform the need for updates of the current SARS-CoV-2 vaccines and help update guidelines suitable for the postpandemic world.

## INTRODUCTION

Severe acute respiratory syndrome coronavirus 2 (SARS-CoV-2) emerged in late 2019 in China and has since caused the coronavirus disease 2019 (COVID-19) pandemic. Early reports from the Wuhan Institute of Virology indicated that individuals infected with this virus mounted antibody responses against it (1). Assays to measure these antibody responses were swiftly developed (2), and the general assumption was that antibodies, especially neutralizing antibodies, would correlate with protection and prevent reinfection. These assumptions were backed up by the detection of neutralizing antibodies after infection (3, 4) and the findings that prior infection protects nonhuman primates from reinfection (5, 6). Importantly, SARS-CoV-2 spike antibodies provided a correlate of protection in the nonhuman primate model (7, 8). However, reports of reinfections (9, 10), reports about waning of antibody within 8 weeks (11) (which turned out to be misleading), and studies suggesting reinfection with human seasonal coronaviruses (hCoVs) (12–14) highlight the need for large longitudinal observational studies to test these assumptions in a rigorous manner.

**FIG 1:**
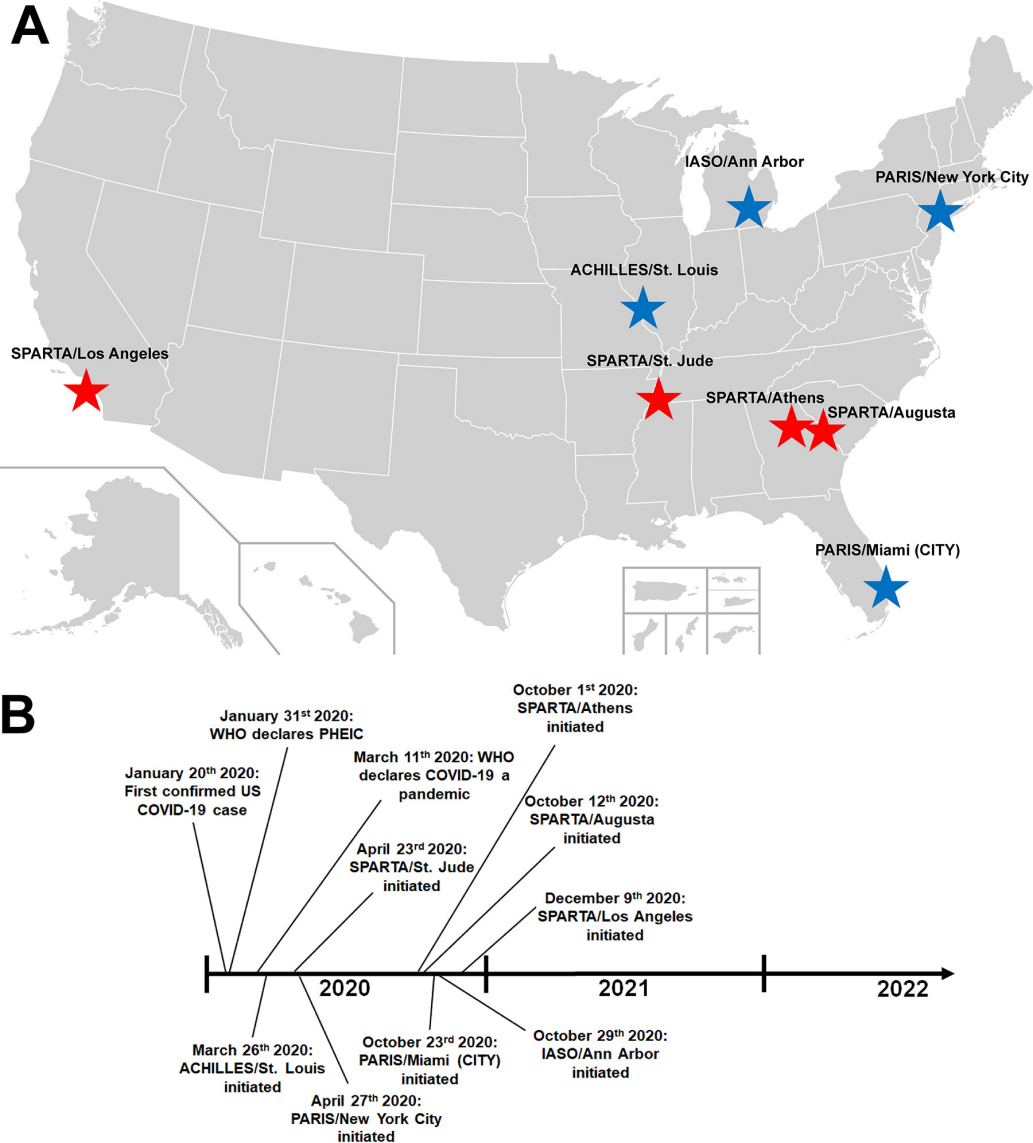
Overview of the PARIS/SPARTA cohorts. (A) Geographic location of the different sites in the United States. Blue stars indicate sites supported by 75N93019C00051, red stars indicate sites supported by 75N93019C00052. (B) Timeline of the pandemic in the United States and the establishment of the different cohorts. The U.S. map in panel A was used as permitted under a CC0 license (source: https://commons.wikimedia.org/wiki/File:Blank_USA,_w_territories.svg).

We established several longitudinal cohorts under the umbrella of the PARIS (Protection Associated with Rapid Immunity to SARS-CoV-2) and SPARTA (SARS SeroPrevalence And Respiratory Tract Assessment) studies to address the question of SARS-CoV-2 antibody durability and efficacy (e.g., protection against reinfection). These human cohorts were initially designed to compare the frequency of SARS-CoV-2 infection in seropositive to seronegative participants, thus pinpointing correlates of protection in the context of natural infection. With the rapid SARS-CoV-2 vaccine rollouts starting in mid-December 2020 in the United States, many of our cohorts also now track immune responses to vaccination in both seronegative and seropositive individuals at the time of immunization. These natural infection/vaccine cohorts will help to establish correlates of protection after natural infection and vaccination and inform about vaccine-induced immunity to newly emerging SARS-CoV-2 variants of interest/concern (15–20) (Fig. 1).

## CONCEPT

The original PARIS cohort was planned and established at the Icahn School of Medicine at Mount Sinai in New York, NY, with the first participants being enrolled on 27 April 2020. During the following months, seven other cohorts were added to provide geographic diversity and achieve more statistical power (Table 1, Fig. 1). These cohorts were either preexisting (St. Jude, Washington University) or newly initiated. The overall concept for all cohorts was to follow individuals with and without prior COVID-19 by collecting data as well as biospecimens to measure immune responses (e.g., antibody responses to the spike protein of SARS-CoV-2) at least every 2 months. Molecular SARS-CoV-2 testing of symptomatic individuals to identify SARS-CoV-2, akin to phase III vaccine trials, was also included in each of the cohorts.

**TABLE 1:** Overview of the PARIS/SPARTA cohorts, status as of 28 February 2022^*a*^

Cohort/location	Target enrollment (current enrollment)	Date of first enrollment	Sex, age, ethnicity distribution	Specimen and sampling intervals	Percent vaccinated	Percent boosted	No. of breakthrough infections after full vaccination	Principal investigator	Studies published
ACHILLES/St. Louis, MO	800 (671)	26 March 2020	49% female Avg age, 54.5 yrs (range, 18.9–93.6) 49% white, 48% African-American	Serum, saliva, PBMCs 1/8 wks	NA	NA	NA	Ali Ellebedy	22, 35, 41–44
SPARTA/St. Jude	1,315 (1,316)	23 April 2020	73% female Avg age, 43.7 yrs (range, 20–83 yrs) 80% white	Plasma, PBMCs 2 and 4 wks post event, then every 3 mo	91	NA	NA	Paul Thomas	36, 45–48
PARIS/New York City	400 (500)	27 April 2020	67% female Avg age, 39 yrs (range, 18–74 yrs) 55% white	Saliva, serum, plasma, PBMCs 2/4 wks	92	81	90	Viviana Simon, Florian Krammer	32–34, 37, 39, 40, 49–51
SPARTA/Athens, GA	1,500 (1,833)	1 October 2020	69% female Avg age, 44.7 yrs (range, 18–86) 86% white	Saliva, serum, PBMCs 2/4 wks	74	42.9	236	Ted M. Ross	31, 52–54
SPARTA/Augusta, GA^*b*^	1,500 (637)	12 October 2020	62% female Avg age, 45 yrs (range, 18–85) 49% white, 38% African-American	Saliva, serum, PBMCs 2/4 wks	55	15	NA	Ravindra Kolhe	55 – 59
PARIS/Miami, FL (CITY)	200 (228)	23 October 2020	60% female Avg age, 48.2 yrs (range, 20–92) 84% white, 6.6% African-American, 38.5% Hispanic/LatinX	Serum, plasma, PBMCs 4/8 wks	87.2	50.8	31	Savita Pahwa	
IASO/Ann Arbor, MI	5,000 (3,356)	29 October 2020	78.1% female Avg age, 44.3 yrs (range, 18–94) 85.9% white	Serum, PBMCs 8 wks	96.4	85.7	385	Aubree Gordon, Riccardo Valdez	60, 61
SPARTA/Los Angeles, CA	200 (200)	9 December 2020	70% female Avg age, 40.2 yrs (range, 19–77) 16.5% white, 29% Asian, 40.5% Hispanic/ LatinX	Saliva, serum, PBMCs 2/4 wks	83	32	23	Elaine Reed	

a NA, Not applicable.

b Status as of 1 April 2022, not 28 February 2022.

A shared theme of all eight cohorts was to enroll individuals who were at greater risk of COVID-19 in order to accumulate new infections faster. The initial PARIS study focused on health care workers (HCWs) in New York City, one of the early epicenters of the pandemic in the United States, but the other cohorts targeted other populations, including communities of color, first responders, and students. All cohorts used the same serological assay, which had originally been developed at Mount Sinai, to measure SARS-CoV-2 spike IgG antibodies (3, 21). This assay is an orthogonal enzyme-linked immunosorbent assay with high specificity and sensitivity which measures immune responses to the SARS-CoV-2 spike protein and therefore captures both infection- and vaccine-induced serum antibody responses (21). A detailed protocol for this assay has been published (21). However, as described below, many sites also collect saliva samples and perform molecular testing for not only SARS-CoV-2 but also other respiratory pathogens using multiplex diagnostic panels, and some cohorts sample and collect data at shorter (e.g., 2- or 4-week) intervals.

The eight PARIS/SPARTA cohorts are geographically distributed across the United States, covering the coasts (New York, NY, Los Angeles, CA, Miami, FL, Augusta, GA, Athens, GA) as well as the heartland (Memphis, TN, St. Louis, MO, Ann Arbor, MI). Collectively, these eight cohorts provide COVID-19-specific information and biospecimens, at minimum, every 2 months for a total of 8,741 participants. While the primary analysis of antibody responses specific for each cohort is conducted by each site, a secondary analysis of the immune response data generated by the eight cohorts using the harmonized collection time points and assays will be performed by the common data analysis site established for the purpose of cross-cohort analysis and data modeling.

## DETAILED COHORT DESCRIPTIONS

### PARIS (NYC).

This cohort follows health care workers from the Mount Sinai Health System in New York City (NYC). Immediate household members were also eligible for participation. The first participants were enrolled on 27 April 2020. A total of 500 participants completed at least one study visit as of 28 February 2022, with 412 currently active participants (withdrawal rate, 18%). In total, 67% of participants are female, and the average age is 39 years (range: minimum [min], 18; maximum [max], 74 years); 55% of the participants self-identify as white, 14% as Asian, and 6% as African-American. In addition, 13% identify as Hispanic/LatinX. A total of 36% of the participants were seropositive at enrollment, with the majority having been infected with SARS-CoV-2 in March/April of 2020 during the first pandemic wave when New York City emerged as one of the early epicenters of the pandemic in the United States. A total of 92% of the cohort has been fully vaccinated with mRNA vaccines (BNT162b2 [Pfizer] or mRNA-1273 [Moderna]), and 81% have received a booster vaccination (as of February 2022). A total of 90 PARIS participants tested positive for SARS-CoV-2 after being fully vaccinated (two doses of mRNA vaccines). At each study visit, saliva, serum, plasma, and peripheral blood mononuclear cells (PBMCs) are collected and cryopreserved. Nasopharyngeal swabbing is performed when participants report signs and symptoms suggestive of upper respiratory tract infections. Full-length spike binding IgG antibody concentrations are measured at each study visit.

### IASO (PARIS/Ann Arbor, MI).

The Immunity Associated with SARS-COV-2 (IASO) study follows staff and student employees at the University of Michigan, Ann Arbor. The first participant was enrolled on 29 October 2020, and as of 28 February 2021, 2,541 participants were active in the study, and enrollment is ongoing. To date, a total of 3,356 participants have ever been enrolled, 300 have withdrawn from the study (withdrawal rate, 8.9%), and 515 (15.3%) participants chose not to enroll in year 2. The average age of participants is 44.3 years (range: min., 18; max., 94 years), and 78.1% are female; 85.9% of the participants self-identify as white, 7.3% as Asian, 2.7% as African-American, 0.01% as Native Hawaiian or other Pacific Islander, 0.1% as American Indian or Alaska Native, and 2.0% as multiracial. In addition, 3.7% identified as Hispanic/LatinX. A total of 96.4% of the cohort was fully vaccinated as of 28 February 2021, and 85.7% had received a booster. Prior to vaccination, 62.8% of participants were SARS-CoV-2 naive, 9.6% were infected, 12.2% were vaccinated with an unknown infection history, and 3.0% were both vaccinated and infected, with timing of infection unknown; 0.5% of vaccinated participants were infected during the course of vaccination (between doses 1 and 2 or <14 days after dose 2), while 11.9% of vaccinated participants experienced a breakthrough infection (positive ≥14 days after dose 2). Of the 385 reported breakthrough infections, 284 (73.8%) were confirmed by PCR. Of study participants that are currently unvaccinated, 30.3% have no infection history and 69.7% have a history of at least one infection with SARS-CoV-2. At each study visit, serum is collected and cryopreserved. Asymptomatic serial respiratory samples and/or and peripheral blood mononuclear cells and plasma are collected from a subset of the cohort.

### ACHILLES (PARIS/St. Louis, MO).

The ACHILLES cohort comprises two study protocols enrolling participants at the Barnes-Jewish Hospital system and at the Infectious Disease Clinical Research Unit at Washington University School of Medicine. The first is the WU-350 study, which is a sample collection study that enrolled participants who were being tested for SARS-CoV-2, and thus, baseline samples consist of both SARS-CoV-2-positive and -negative participants. Participants who test positive for SARS-CoV-2 are subsequently followed at 3, 7, 14, and 28 days and then 3, 6, 9, and 12 months after infection. Blood, saliva, and other biospecimens are collected from these participants where possible. In total, 500 participants were enrolled in WU-350. This cohort consists of 233 women and 267 men, with 314 African-American 177 white, and 9 Asian participants. The second study, WU-353, enrolled participants with PCR-confirmed SARS-CoV-2 infection who were convalescent and had been tested positive at least 14 days prior to the baseline visit. These participants are also followed every 3 months for 2 years. A total of 171 participants were enrolled in this study. Current demographics are 97 women and 73 men and 1 undisclosed, with 154 white participants, 7 Asian, 6 African-American, and 4 with more than one race or unreported. These participants provided samples every 3 months for up to 2 years. We have also enrolled several of these participants in substudies, which collect lymph node or bone marrow biopsy specimens. Information is being collected from both cohorts about symptoms, recurrent infections, and vaccinations (22).

### CITY (PARIS/Miami, FL).

The COVID Immunity Study (CITY) cohort follows high-risk groups such as health care workers and participants from the community, with a target enrollment of 200 participants divided between those with confirmed prior SARS-CoV-2 infection or self-reported as uninfected. The first participant was enrolled on 23 October 2020, and enrollment is complete. As of 18 November 2021, we have recruited 228 participants (39.9% males and 60.1% females) with an average age of 48.2 years (range: min., 20; max., 92 years). A total of 84% of the participants self-identified as white, 6.6% as African-American, 5.2% as Asian, and 3.9% as other. Overall, 38.5% identified as Hispanic/LatinX. SARS-CoV-2 infection status is well matched, with 49.5% (113) having had SARS-CoV-2 infection and 50.5% (115) not ever being SARS-CoV-2 infected. A total of 87.2% (199) of the cohort has received at least one dose of an mRNA-vaccine (BNT162b2 or mRNA-1273) or vectored vaccine (J&J). Based on their real-time study status, only 6% of the SARS-CoV-2-uninfected group and 21.2% of those with past COVID-19 diagnosis have not yet been vaccinated. Participants are followed monthly for the first 6 visits, including baseline, and then every other month for each subsequent visit up to 2 years; 118 participants have completed year 1 and are continuing into year 2. At each visit, serum, plasma, and peripheral blood mononuclear cells are collected and cryopreserved. Overall study withdrawal occurred in 20.2% (46) of participants; of these, 45.6% (21) were in the noninfected and 54.3% (25) in the previously infected group. In addition to the regular follow-ups, we are collecting samples after COVID-19 vaccine and booster doses at two additional time points (1 week and 1 month post-last vaccine dose and booster) to collect plasma, serum, and PBMCs. At present, we have these samples collected from 84 participants.

### SPARTA/Athens.

The SPARTA/Athens study follows university employees and students at the University of Georgia, hospital workers from Piedmont Athens Regional and St. Mary’s Hospitals, first responders, and residents from the local Atlanta, GA, and Athens, GA, communities. The first participant was enrolled on 1 October 2020. As of 28 February 2022, 1,833 participants were enrolled in the study, and enrollment is ongoing. Of the potential participants that completed a study screening call, 1,833 enrolled, 60 declined enrollment, and the enrollment process is ongoing for 10 participants. To date, 114 participants have withdrawn from the study (withdrawal rate, 6.5%). A total of 69% of participants are female, 31% are male, and 0.3% identify as other. The average age is 44.7 years (range: min., 18; max., 86 years); 86% of the participants self-identify as white, 4% as Asian, 7% as African-American, and 2% as other or multiple. In addition, 6% identified as Hispanic/LatinX. A total of 13% of participants were previously infected at enrollment, and 61% were SARS-CoV-2 naive; 74% of the cohort was vaccinated as of 28 February 2022, and 61% were vaccinated without history of infection. A total of 931 participants received the BNT162b2 (Pfizer)vaccine, 383 participants received the mRNA-1273 (Moderna) vaccine, 3 participants received the AZD1222 (AstraZeneca) vaccine, and 29 participants received the Ad26.COV2.S (J&J) vaccine; 42.9% of the entire Athens cohort (786 out of 1,833 total participants) received a vaccine booster. A total of 236 breakthrough infections occurred as of February 2022 in the Athens cohort. Of the Athens/SPARTA participants, 11% (175) are currently antibody negative and 89% (1,559) are antibody positive, largely due to vaccination. There are 33 participants that have tested positive for viral RNA following nasal swab/saliva collection, which amounts to 2% of the Athens/SPARTA cohort. At each visit, saliva, serum, plasma, and peripheral blood mononuclear cells are collected and cryopreserved.

### SPARTA/Augusta.

The SPARTA/Augusta study follows Augusta University Medical Center health care workers and students at the Augusta University, hospital workers from the Dental College of Georgia, and residents from the regional Central Savannah River Area communities. The first participant was enrolled on 12 October 2020. As of 1 April 2022, 637 participants were enrolled in the study, and enrollment is ongoing. To date, 108 participants have formally withdrawn or transferred to another study site. Of all subjects enrolled, 62.1% are female and 37.9% are male. The average age is ~45 years (range, 18 to 85). A total of 49.1% self-identify as white, 10.7% as Asian, 38.2% as African-American, and 2.6% as other or multiple, and 5.2% identify as Hispanic/LatinX. In addition, 49.9% were previously infected or vaccinated at enrollment, and 51.1% were SARS-CoV-2 naive. 177 participants tested positive at enrollment for viral RNA following nasal swab/saliva collection, which is 30% of the cohort. Currently, 25.4% are antibody negative and 74.1% are antibody positive, largely due to vaccination. At each visit, saliva, serum, plasma, and peripheral blood mononuclear cells are collected and cryopreserved.

### SPARTA/St. Jude.

The SPARTA/St. Jude (SJTRC) study follows hospital employees, with both direct and indirect patient contact, at St. Jude Children’s Research Hospital (SJCRH) in Memphis, TN. The first participant was enrolled on 23 April 2020, and of the potential participants who received the study participation email, 1,316 had enrolled as of 28 February 2022. Enrollment is closed for the naive arm of the study but is ongoing for infected individuals. To date, 80 participants have withdrawn from the study, mostly because they no longer work at SJCRH (withdrawal rate, 6%). In all, 73.4% of participants are female, and 26.3% are male. The average age is 43.7 years (range: min., 20; max., 83 years). A total of 80% of the participants self-identified as white, 9% as Asian, 8.1% as African-American, 0.1% as American Indian/Alaska Native, and 2.6% as other or declined to answer. In addition, 3.6% identified as Hispanic/LatinX, and 94.7% identified as non-Hispanic. As of 28 February 2022, of the unvaccinated participants, 6.7% (88) had no infection history, and 2.4% (31) have infection history. Of the vaccinated participants, 77.9% (1,025) have no infection history, 10.6% (139) were infected prior to vaccination, 1.0% (13) were infected during vaccination or <14 days following the full course of vaccination, and 1.9% (25) were infected ≥14 days following the full course of vaccination. A total of 1,049 participants received BNT162b2 (Pfizer), 135 participants received mRNA-1273 (Moderna), 17 participants received Ad26.COV2.S (J&J), and 1 participant received multiple vaccines. Of the SJTRC participants with serological tests run, 926 individuals are SARS-CoV-2 receptor binding domain (RBD)- and spike IgG antibody-positive, largely due to vaccination. There are 192 participants that have tested positive for viral RNA following nasal swab/saliva collection, which is 14.6% of the SJTRC cohort. Subjects are routinely swabbed as part of an employee screening program and have consented to have those results and their swabs used for study purposes. Symptomatic screening is also performed. For baseline enrollment, plasma and peripheral blood mononuclear cells are collected and cryopreserved. Following a positive PCR test, plasma and peripheral blood mononuclear cells are collected and cryopreserved at acute (<day 14), convalescent (~day 28,) and postconvalescent (every subsequent 3 months) time points. Postvaccine time points (approximately day 14 after completion) are also collected for plasma and peripheral blood mononuclear cells.

### SPARTA/Los Angeles, CA.

The Los Angeles-SPARTA (SPARTA/LA) component of the program is the western-most site of the PARIS/SPARTA network. SPARTA/LA is based at the Lundquist Institute on the Harbor-UCLA Medical Center Campus in southwest Los Angeles County. Participant enrollment was focused on individuals at the intersection of a real-world situation of great concern—high COVID-19 risk and high vaccine hesitancy. In this respect, the recruitment goal was to engage participants likely to have a high risk of SARS-CoV-2 exposure but who represent populations less likely to be vaccinated, including Hispanic/LatinX, Pacific Islanders, and African-American and other underrepresented groups. Participants were enrolled based on results of viral nucleic acid and/or antibody testing and then followed in one of two parallel study cohorts; cohort A followed individuals with documented acute SARS-CoV-2 infection (PCR positive for SARS-CoV-2 RNA) at time of enrollment, and cohort B followed individuals found to be antibody positive due to prior infection or vaccination. The first participant was enrolled on 9 December 2020. As of 28 February 2022, 200 participants were enrolled in the study, and enrollment is complete. To date, 12 participants (6%) have withdrawn from the study. Among enrolled participants, 70% are female and 30% are male. The mean age of participants is 40.2 years (range, 19 to 77). Racial/ethnic background was 40.5% Hispanic/LatinX, 29% Asian, 16.5% non-Hispanic white, 6.5% African-American, 4% mixed race, 2% Native Hawaiian/Pacific Islander, 0.5% American Indian/Alaskan Native, and 1% other/declined to answer. The vaccination rate in our cohort was 83% as of 28 February 2022; 83% of vaccinated participants received the BNT162b2 (Pfizer) vaccine, 15% received the mRNA-1273 (Moderna) vaccine, and 2% received the Ad26.COV2.S (J&J) vaccine. At enrollment, 18% were antibody negative and 82% were antibody positive (65.5% due to vaccination and 16.5% due to prior SARS-CoV-2 infection). At each study visit, saliva, serum, plasma, and peripheral blood mononuclear cells are collected and cryopreserved.

## PARIS/SPARTA INFECTION AND REINFECTION CLASSIFICATION

An important aspect of the PARIS and SPARTA cohorts is the common unified definitions of infection and reinfection, which are applicable both in the pre- as well as postvaccine era. Of note, these temporary working definitions will be continuously refined as we obtain more data from our longitudinal cohorts and as understanding of the immunological and virological dynamics of SARS-CoV-2 infections grows. Our current classification includes distinct categories for seronegative versus seropositive individuals. Factors taken into account are the reliability of diagnostic molecular and serological tests, the time between potential initial infection and reinfection, complete viral genome sequence information, and consistent increase in serum antibody responses. Missing/unobserved data will be taken into account in the future.

### SARS-COV-2 infection in a previously seronegative individual.

**(i) Possible infection.** One positive test including any of the following: nucleic acid amplification test (NAAT), rapid antigen test, or serological test (e.g., SARS-CoV-2 IgM/IgG).

**(ii) Confirmed infection.** A combination of any two positive tests completed, including molecular tests (NAAT), rapid antigen tests, complete viral genome sequencing, clinical diagnosis of COVID-19, or serological assays used in the study (3, 21). With the exception of complete viral genome sequencing, diagnostic tests must be completed on a different biospecimen.

### SARS-COV-2 infection in a seropositive individual. (established seroconverted due to infection or vaccination). (i) Possible reinfection.

Possible reinfections need to meet one of the following scenarios: (i) Two positive molecular tests (NAAT) at least 90 days apart. (ii) A stable (two measurements at a lower level followed by two measurements at a higher level) 4-fold increase in serum spike antibody titer in individuals who are seropositive. (iii) The combination of a documented positive NAAT for the primary infection and evidence for the second infection being caused by a viral strain that did not exist/circulate in a given region at the time of the first infection (and an unlikely within-host descendant of the previously infecting strain) is also considered as representing a likely reinfection.

**(ii) Possible breakthrough infection after vaccination.** One positive test including any of the following: NAAT, rapid antigen test, or serological test against nucleoprotein.

**(iii) Confirmed reinfection.** Two NAAT-confirmed SARS-CoV-2 infections in which both viruses were isolated, sequenced, and determined to be phylogenetically distinct to constitute separate infection events.

**(iv) Confirmed breakthrough infection after vaccination.** A combination of any two positive tests, including molecular tests (NAAT), rapid antigen tests, complete viral genome sequencing conducted on a different biospecimen, serological test against nucleoprotein, or clinical diagnosis of COVID-19. A change in antibody levels after the infection is also indicative of an infection. Of note, individuals who are seropositive exclusively due to vaccination, will not develop antibodies to regions other than spike (e.g., N protein).

## DISCUSSION

We established the PARIS and SPARTA cohorts to study infection with SARS-CoV-2 in seropositive and seronegative participants at high risk for infection. In addition to defining the durability and variability of antibody responses, we seek to obtain data on the attack rates in both groups, which will inform about the risk of reinfection and help identify the spike antibody titers that correlate with protection in individuals with different histories of exposure to SARS-CoV-2.

The data collected across the studies (some of which started as early as March/April 2020) will provide insights into infection and reinfection with SARS-CoV-2 (23–30) in naive and vaccinated participants, including by variants of concern, once the ongoing analysis is completed.

Many of the PARIS/SPARTA participants are health care workers, who were first in line to be eligible for SARS-CoV-2 vaccination starting in December 2020 and who were also first in line for booster doses in 2021. Thus, vaccination rates in several of the cohorts increased rapidly leading to the conversion of the initial natural infection studies to studies that are suited to characterize the protective effects of vaccines, including immune responses mounted to vaccination in naive individuals and individuals who previously had COVID-19, immune responses to booster doses and breakthrough infections as well as vaccine-induced correlates of protection (31, 32). With the emergence of viral variants of interest/concern, the PARIS/SPARTA cohorts started also to characterize viruses isolated from participants infected after vaccination to determine if viral variants carrying mutations indicative of immune escape are present. To date, data and samples from PARIS/SPARTA have already been used in several studies, including work that determined that only one SARS-CoV-2 mRNA vaccine shot may be necessary in individuals previously infected with COVID-19 (see references to PARIS/SPARTA manuscripts in Table 1). The data collected from PARIS/SPARTA participants also have value as a healthy control group, which can be used as a reference when analyzing immune responses observed in patient populations with specific immune-modulatory comorbidities (e.g., posttransplant patients, patients with B cell malignancies, or patients receiving treatment with biologicals) (33, 34). In addition, cohort samples have been a rich source to study the longevity of infection-induced B-cells in the bone marrow, to study affinity maturation of B-cells after COVID-19 vaccination, to characterize the T-cell response to SARS-CoV-2, etc., and similar studies using PARIS/SPARTA samples are ongoing (22, 35, 36). Currently, samples from PARIS/SPARTA are being extensively used to determine immune responses after booster doses and after breakthrough infections, and assessment of mucosal immune responses in the cohorts is also ongoing (37). Furthermore, samples from the PARIS/SPARTA cohorts have been shared with and used extensively by the SARS-CoV-2 Assessment of Viral Evolution (SAVE) program (38), which was established by the National Institutes of Health to track and characterize variants of concern (39, 40). SAVE investigators have used these samples to track immune escape of different variants from neutralizing antibodies (38).

This study design is unique in that it encompasses eight geographically distinct cohorts exploring specific aspects of viral infections and vaccination using harmonized assays and analysis, which allows for pooling of data from many study participants for robust secondary analysis. However, one limitation is, that the study population consists of mostly health care workers and is in general healthy and younger, with individuals with comorbidities being underrepresented. In the near future, the PARIS/SPARTA cohorts will be uniquely suited to track the kinetics of and protection afforded by vaccine-induced immunity over time, including against novel viral variants, and to investigate how vaccination influences postacute sequelae of COVID-19 (PASC, long COVID-19).
